# The Assessment of Sperm DNA Integrity: Implications for Assisted Reproductive Technology Fertility Outcomes across Livestock Species

**DOI:** 10.3390/biology13070539

**Published:** 2024-07-17

**Authors:** Maya J. Robertson, Caitlin Chambers, Eloise A. Spanner, Simon P. de Graaf, Jessica P. Rickard

**Affiliations:** Faculty of Science, School of Life and Environmental Sciences, The University of Sydney, Sydney, NSW 2006, Australia; mrob6931@uni.sydney.edu.au (M.J.R.); simon.degraaf@sydney.edu.au (S.P.d.G.)

**Keywords:** DNA, chromatin, fertility, artificial reproductive technologies, livestock, sperm

## Abstract

**Simple Summary:**

This review explores the importance of sperm DNA integrity in assisted reproductive technologies used in both livestock production and human fertility treatments. Assisted reproductive technologies have revolutionised animal breeding by enabling the widespread dissemination of elite male genetics and overcoming human infertility challenges, with male-factor infertility being a significant concern. Traditional semen quality assessments focus on parameters like volume, concentration, motility and morphology; however, DNA integrity, a potential key parameter in assessing semen quality, is not included despite being studied for many years. Various assays are used to measure sperm DNA fragmentation, but results on its impact on fertility are inconsistent, resulting in a lack of standardisation of assessment methodology. This review consolidates findings across species and DNA integrity assays into a comprehensive table, highlighting DNA integrity as a potential biomarker for male infertility and predictive value for assisted reproductive technology outcomes in livestock and humans.

**Abstract:**

Sperm DNA integrity is increasingly considered a useful measure of semen quality in mammalian reproduction. However, the definition of DNA integrity, the ideal means by which it should be measured, and its predictive value for fertility remain a topic of much discussion. With an emphasis on livestock species, this review discusses the assays that have been developed to measure DNA integrity as well as their correlation with in vitro and in vivo fertility.

## 1. Introduction

Assisted reproductive technologies (ARTs) are frequently used in both livestock production and human fertility treatment. In animal industries, these technologies have allowed widespread dissemination of elite genetics, contributing to advancements in breeding programmes and production objectives. Selecting high-quality sires and semen is crucial for ART success in animal industries, as semen from one male is often used to inseminate multiple females. ARTs also play a crucial role in helping to overcome human infertility. Notably, male-factor infertility has been identified to be partially or completely responsible for half of all infertility cases [1], highlighting the importance of understanding the link between semen quality and successful pregnancy outcomes following ARTs. 

Semen quality is the usual means by which the potential fertility of a male is assessed. The evaluation of semen quality, whether post-collection or post-thaw before insemination, routinely involves assessing parameters such as sample volume, concentration, motility, and morphology [2,3]. Continued research efforts have attempted to standardise the assessment of these semen factors, leading to the development of more objective analysis techniques [2] such as the use of a computer-assisted sperm analyser (CASA) for sperm motility and kinematics [4] and the introduction of new technologies allowing the exploration of sperm DNA integrity.

The integrity of a sperm genome is a fundamental factor in the development of healthy offspring and can be an effective diagnostic tool for sperm reproductive potential [5]. Driven by histone substitution by protamine, genome formation occurs in the sperm nucleus during testicular development and epididymal maturation. This process causes chromatin remodelling, forming DNA into toroidal structures [6]. Intact DNA is defined as ‘the complete absence of nicks and breaks, either single or double-stranded, or any chemical modifications in its structure’ [7]. Sperm DNA fragmentation (SDF) is the accumulation of single- (SSBs) or double-stranded breaks (DSBs), measured by the DNA Fragmentation Index (DFI). DFI is calculated by the number of single-stranded DNA (ss-DNA) in a sample divided by the total number of spermatozoa with intact DNA expressed as a percentage [5].

While the impact of other semen factors on fertility potential has been investigated across various species [8,9,10], conclusive and uniform findings regarding the influence of DNA integrity remain elusive. DNA damage to the sperm cell can be measured using various assays, which has likely contributed to the reporting of diverse outcomes on its impact on fertility, posing challenges in comparing trends across and within species. Common assays used to estimate sperm DNA damage across species include but are not limited to Sperm Chromatin Structure Assay (SCSA), Sperm Chromatin Dispersion test (SCD), Transferase dUTP Nick End Labelling (TUNEL) and single-cell gel electrophoresis (COMET). While certain studies have highlighted a potential connection between sperm DNA fragmentation and fertility in bulls [11,12], stallions [13], humans [14], boars [15], and rams [16,17], conflicting results have also been reported on the same species [18,19,20]. Collating results between assays and particular species could help establish whether there are any species-specific or sperm-type-specific trends related to particular assay use. It also emphasises a notable gap where the analysis of DNA integrity could enhance our understanding of in vitro semen quality and fertility in livestock species. 

This review aims to cover commonly used methods of sperm DNA integrity assessment and their implications for ART outcomes across both livestock species and humans. Through this exploration, we aim to comprehensively analyse DNA integrity as a semen quality parameter, examining its potential as a biomarker for male infertility and its ability to enhance predictive accuracy for ART outcomes in both production animals and humans. 

## 2. DNA Maturation and the Development of Spermatozoa 

The process of sperm development, DNA compaction and maturation is a complex and biologically distinct three-step process: mitosis, meiosis and spermiogenesis. The following section will outline the major processes in this developmental journey and how interruptions or mutations can occur. The potential sources of DNA damage will be discussed, and how this influences the ability of spermatozoa to achieve fertilisation under both natural and artificial conditions.

### 2.1. Testicular Development 

**Figure 1:**
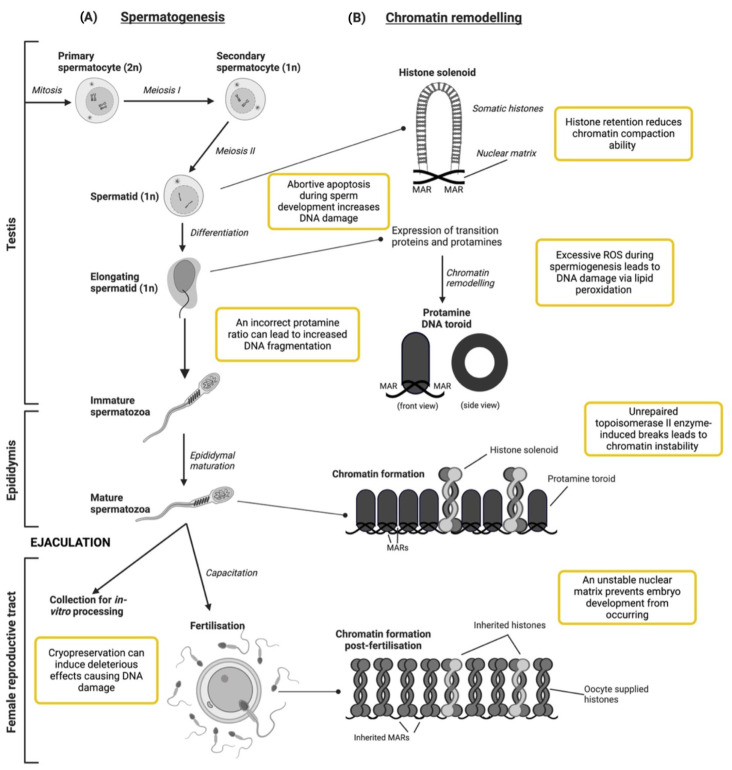
An overview of sperm development and chromatin dynamics during histone–protamine exchange. (**A**) Spermatocytes undergo mitotic division to form haploid cells, which then undergo meiosis to form spermatids. Ejaculated sperm can be collected for in vitro processing or be deposited into the female reproductive tract, where they undergo the capacitation and acrosome reaction [21], enabling penetration of the oocyte zone pellucida via the acrosome. (**B**) As chromatin compaction progresses during testicular development and epididymal maturation, the nucleosomal architecture shifts to a toroidal structure facilitated by sperm nuclear basic proteins. During spermatid elongation, transitional proteins are expressed, leading to histone replacement by protamine proteins. This results in tightly packaged toroids of DNA attached to the sperm nuclear MAR stacked side by side with a small number of retained histones. Following fertilisation, sperm-specific protamines are replaced with oocyte-supplied histones. Histone-bound chromatin and MARs from the sperm are retained in the newly formed pronucleus, altering chromatin accessibility. The yellow boxes denote potential causes of DNA damage or embryo development disruptions throughout these processes. Figure created with BioRender.com. This protamine-bound chromatin structure is a model of the most efficient form toroids could condense to, based on a figure published by Ward, 2009 [23]. Abbreviations: DNA, deoxynucleic acid; MAR, matrix attachment region.

The physiological development of spermatozoa begins in foetal life, with primordial germ cells forming stem-cell spermatogonia within the testis [21]. Once an animal reaches puberty, spermatogenesis commences, involving mitotic proliferation and meiotic division of spermatogonia, which halves the DNA content, creating haploid spermatids. Chromatin undergoes alterations throughout meiosis and spermiogenesis, where spermatids elongate to form mature sperm with tightly compacted chromatin (Figure 1). Spermatid transformation can be divided into four phases: the Golgi, cap, acrosomal and maturation phases [22]. During these phases, the spermatid undergoes significant structural changes, including the formation of the acrosomal vesicle in the Golgi phase and the acrosomal cap during the cap and acrosomal phase [22]. During both the acrosomal and maturation phases, extensive chromatin remodelling occurs to shape the nucleus into a flattened structure. 

The chromatin remodelling process from histone to protamine, known as protamination, shown in Figure 1, is one of the more poorly understood processes of spermiogenesis. Chromatin, the basic unit of which is the nucleosome, comprises 146 base pairs of DNA wrapped around a core histone octamer [24]. Chromatin compaction is initiated by somatic histones in developing sperm being replaced by sperm nuclear basic proteins. These proteins include testis-expressed histone subunits, transitional nuclear proteins (TPs) and protamine proteins [6]. It was previously understood that protamination occurred in a step-wise fashion [25], but current research indicates that histones and transitional nuclear proteins co-occur, driving the recruitment and processing of protamines [26]. 

Presumed to regulate the compaction process, TPs are only known to be involved in certain species, including humans, rodents, boars and rams [27,28,29]. The major TPs include TP1, which is highly expressed, and TP2, which is poorly conserved. Beyond being a mediator protein between histones and protamines, their importance is not conclusively known. A study using mutant mice without TP1 revealed successful sperm production with limited abnormalities, suggesting the potential redundancy of TPs in the development process [30]. However, double-knockout mice were completely infertile, indicating the critical role of TPs in sperm chromatin composition and development [31]. Regardless, it is understood that protamines replace TPs during spermatid elongation [6,26]. 

Protamines are small basic proteins exclusively found in mature spermatozoa [32]. There are two forms of protamines: Protamine 1 (P1) is expressed in its mature form in all mammals, whereas Protamine 2 (P2), which matures once bound to DNA, is expressed by some mammalian species, including man, mice and stallions [32]. The correct P1:P2 ratio within species is crucial for normal sperm development, as deviations have been linked to increased DNA fragmentation in mice and humans [33,34,35]. Although protamines ultimately displace the majority of histones during spermiogenesis, there is still 2 to 15% of mammalian chromatin, depending on the species and experiment [36,37,38], bound to histones after compaction that is associated and bound to specific genes at gene protomer regions on the nuclear matrix [39] (Figure 1).

Protamines are characterised by their arginine-rich DNA-anchoring domains and cysteine-rich sequences [32]. The anchoring domains assist in binding protamine to the DNA backbone, while the cysteine-rich sequences facilitate the formation of multiple di-sulphide bonds and zinc bridges between protamines, ultimately bending the DNA into a toroidal structure [25,40]. Connecting each toroid structure are nuclear-sensitive segments of chromatin called toroid linkers, which are also the site of attachment of DNA to the nuclear matrix or matrix attachment regions (MARs) [41]. The hydrodynamic shape visualised in Figure 1 offers the greatest protection to the paternal DNA during transit to the egg, as studies have found that protamine–DNA compaction provides protection from radiation damage [42,43]. The nuclear matrix is considered a checkpoint for DNA integrity, as embryo development cannot occur without MAR organisation and an intact matrix [44].

Ultrastructural changes continue in the maturation stage of spermatid transformation as the fibrous sheath that covers the axoneme develops, the mitochondria become tightly packed along the mid-piece, and excess cytoplasm is shed [45]. The elongated spermatids are then ready to be released into the lumen of the tubules and move along the rete testis as spermatozoa. In most mammals, the total duration of spermatogenesis lasts 40 to 54 days in the testis [46]. In humans, the spermatogenic cycle lasts more than 70 days and differs from other common mammals by the low number of sperm produced per gram of testis [47]. 

#### Susceptibility of Chromatin to Damage during Testicular Development 

Although several mechanisms highlighted in Figure 1 are known to cause DNA damage or disrupt sperm maturation during spermiogenesis, the exact source of DNA damage is not yet well understood. As previously mentioned, a correct protamine ratio and intact nuclear matrix are essential for normal sperm development. Any abnormalities in chromatin compaction may manifest in irregularities in the acrosome shape [22]. Sperm head abnormalities are one of the most common morphological defects linked to fertility across species, as the integrity of the acrosome and sperm head must be maintained for the spermatozoa to bind to the oocyte’s zona pellucida for fertilisation [48]. In addition, there are other points throughout sperm development and in vitro processing where DNA is highly susceptible to damage. 

The formation of disulphide bridges between protamines is aided by reactive oxygen species (ROS), ensuring chromatin stability and DNA protection [49,50]. ROS, including all free radicals with an oxygen atom, are generated endogenously by sperm mostly from electron leakage from the mitochondria or enzymes in their plasma membrane. They play pivotal roles in mediating physiological sperm functions such as maturation, motility, capacitation and sperm–oocyte fusion [51,52,53]. However, disruption in spermiogenesis, increasing the number of immature spermatozoa with distorted morphology or cytoplasmic retention, can elevate ROS levels. Excessive ROS can lead to lipid peroxidation of the sperm plasma membrane, compromising sperm functions such as DNA integrity [54]. This oxidative stress-induced DNA damage accelerates apoptosis, ultimately reducing sperm counts. Higher levels of apoptosis, correlated with ROS levels, have been observed in mature spermatozoa from infertile patients compared to normal sperm donors [55]. Additionally, the phenomenon of ‘abortive apoptosis’ occurs when spermatozoa with apoptotic markers, such as abnormal morphology or nuclear DNA damage, fail to be eliminated, thus increasing the level of damaged sperm [56]. 

Chromatin remodelling also includes a sensitive step where the enzyme, topoisomerase II, causes small breaks in the DNA during spermiogenesis, enabling the compact genetic structure within mature sperm to be formed [57]. These breaks must be repaired before the end of the protamination process to avoid the presence of DNA damage in the ejaculate [58]. If this enzyme and process fails, this leads to increased histone retention in mature sperm [59], inadequate protamination and poor chromatin condensation [60]. Consequently, the less compact chromatin structure results in reduced DNA protection, making it more susceptible to damage from intrinsic or extrinsic ROS, particularly during transit through the epididymis [14,61]. Following ejaculation, high ROS levels in the female reproductive tract can also cause DNA damage. If collected artificially for use in ARTs, chromatin damage can also occur throughout in vitro processing. Both scenarios will be discussed further in the following section.

### 2.2. Epididymal Maturation and Fertilisation within the Female Reproductive Tract 

The mammalian epididymis exhibits a consistent structural pattern across mammalian species, with subtle distinctions in its sections and sperm transit times. As the spermatozoon leaves the testis, its motility and ability to fertilise will begin to develop as it transits the epididymis tubule [62]. 

The epididymis is responsible for supplying an optimal environment for the functional maturation of spermatozoa and their storage until ejaculation [62]. Traits like motility and oocyte recognition begin to develop in the caput epididymis and advance, through the corpus before reaching their peak efficiency in the distal caudal segment. As the sperm cells transit the epididymis, the formation of disulphide cross-links increases, further compacting the chromatin into a toroid shape [63,64]. The toroidal configuration optimises compaction, providing greater DNA protection from mechanical disruption than somatic cells [45]. Once fully matured, spermatozoa reside in the cauda until ejaculation, where they are expelled through the vas deferens into the urethra as semen for deposition in the female or collected for in vitro processing. 

Once spermatozoa enter the female reproductive tract, sperm capacitation is initiated by low levels of ROS [65]. This process alters the sperm cellular membrane to ensure penetration of the oocyte and binding to the zona pellucida can occur. However, as previously noted, this process can lead to DNA damage if there are high levels of ROS present [14] (Figure 1). Shortly after fertilisation, sperm-specific protamines are replaced by oocyte-supplied histones, while the original histones bound to chromatin are retained shown in Figure 1 [66]. The primary role of protamines is fertilisation, not embryonic development, as evidenced by their histone replacement 2–4 h after fertilisation [67,68]. Further evidence is shown in a study where round spermatids lacking protamines injected into mouse oocytes demonstrated normal foetal development [69], indicating protamines are not necessary for embryogenesis but play a protective role in ensuring fertilisation. The transfer of a histone-based chromatin structural organisation from sperm to newly fertilised oocytes is crucial for successful embryogenesis [70,71].

The collection and processing of semen for ARTs, such as during freezing and thawing, can have negative consequences on the quality of spermatozoa post-thaw. In vitro processing can increase morphological abnormalities, reducing DNA integrity, viability and fertility outcomes [72,73,74], arising from temperature shock, osmotic stress and ice crystal formation [74,75,76]. Having a thorough understanding of the consequences of in vitro processing on sperm DNA fragmentation or integrity is critical should it be considered as a viable tool to predict sperm fertility following ARTs. 

## 3. Current Measures of DNA Integrity Used across Species 

Across several decades, various methodologies have been employed to assess the DNA integrity of spermatozoa, encompassing a diverse array of assays summarised in Table 1. The earliest methods of assessing sperm DNA integrity date back to the 1980s, with Evenson and Jost developing the Sperm Chromatin Structure Assay (SCSA) [77]. Soon after, the COMET assay, also known as single-cell gel electrophoresis, was introduced [78,79] to measure the extent of DNA damage in sperm cells. The 8-Hydroxyguanine (8-oxoG) assay was introduced soon after this as a way to measure oxidative DNA damage in various biological fluids [80]. Since then, various methods have been developed to assess sperm DNA integrity, summarised in Table 1, including TUNEL [81], Chromomycin A3 test (CMA_3_) [60], toluidine blue (TB) [82] and HALO (Halo Assay for Low-level DNA Fragmentation) [83,84] or SCD [85]. A summary of findings from studies using these various DNA integrity assays to determine the impact of DNA fragmentation on fertility is presented in Table 2. The following section defines each assay and delves into their current use in research applications, critically evaluating their potential to be integrated into the semen assessment toolbox by assessing the influence of sperm DNA integrity on fertility across different species.

### 3.1. Sperm Chromatin Structure Assay (SCSA) 

The flow cytometric SCSA is a diagnostic tool used to measure the susceptibility of sperm nuclear DNA to acid-induced denaturation in situ, which has been correlated with the presence of DNA strand breaks [17,121]. This is achieved by exposing the spermatozoa to an acid–detergent solution, prompting DNA denaturation at the sites of SSBs or DSBs [122]. This assessment can be quantified using flow cytometry, where the semen samples contain a percentage of mature cell DFI that is sorted into either detectable, sperm with increased chromatin damage, or non-detectable groups [123]. Acridine orange (AO) is utilised for its metachromatic properties, where red fluorescence indicates single-stranded, denatured DNA, while green indicates double-stranded, intact DNA using SCSA software (SCSA-Soft2.0; SCSA Diagnostics, Inc., Brookings, SD, USA) [17,123,124]. The ratio of total denaturation is calculated for each spermatozoon in a sample, and the results are expressed as the percentage SDF of cells with high denaturation ratio values [12].

SCSA is well regarded for its efficiency in determining results using flow cytometry as described in Table 1. SCSA results are independent descriptors of semen quality that complement traditional assessments of sperm quality, as parameters are weakly to moderately correlated to concentration, motility and morphology [125]. However, the parameters tested within the SCSA correlate with DNA strand breaks [126] and fertility in vivo [127]. The SCSA can determine the importance of DNA structure in outcomes of ARTs such as AI. 

SCSA has been tested and contrasted in different species, showing high repeatability and sensitivity (Table 2). Flow cytometry-based SCSA has been identified as the most objective and statically robust measure in human fertility clinics [122]. A large human study (*n* = 2262) described in Table 2 found that the early abortion rate increased in the High-DFI (27.3%) and Medium-DFI (14.6%) groups compared to the Low-DFI (4.9%) group, but there was no effect on pregnancy [101]. In another study with 1316 human patients, %DFI was found to be a predictor of pregnancy outcome, with %DFI higher in the non-pregnant group than the pregnant group [102]. As shown in Table 2, SCSA is currently the most commonly used DNA assay in research, especially for animals like bulls. Multiple studies on Norwegian [104], Swedish [11,128], and Holstein bulls [105,106,107,129] have found %DFI to be negatively correlated with non-return rate (NRR) at 56 days. %DFI was also found to negatively affect NRR after 25 days in a study on 15 rams [16]. Some studies identified no significant predictive ability of DNA integrity, measured as %DFI or SDF, for pregnancy or in vitro fertilisation (IVF) outcomes in humans [103,130], boars [20] and stallions [109] (Table 2). As SCSA requires specialised equipment, flow cytometry, and technical expertise to understand clinical significance, there can be difficulties with interpreting the results accurately (Table 1). However, the consistent negative correlation reported in the earlier mentioned studies across several species indicates the soundness of SCSA as an appropriate assay to measure DNA integrity to give insight into fertility outcomes when using ARTs.

### 3.2. Single-Cell Gel Electrophoresis (COMET) 

The COMET assay determines DNA fragmentation by assessing the level of SSBs and/or DSBs in a cell following steps of agarose cell suspension, cell lysis, DNA denaturation (alkaline comet assay only), electrophoresis and microscopy. DNA loops migrate from a damaged cell to the anode [131], forming a COMET tail of fragmented DNA from the nucleoid in which the fluorescence intensity is proportional to the degree of DNA fragmentation [132]. Unlike SCD or TUNEL, COMET can also be quantified to determine each cell’s true degree of DNA fragmentation. COMET tail DNA percentage intensity determines the total amount of fragmented DNA compared to all DNA in a single cell (tail DNA ÷ (tail DNA + head DNA)). COMET tail length is a measure of the size of DNA fragments, as smaller fragments will migrate further than larger fragments during electrophoresis. COMET tail moment is the product of both tail length and tail DNA percentage intensity (tail length × tail DNA %), and finally, the olive tail moment is the product of the percentage of total DNA in the tail and the distance between the centres of the mass of head and tail regions ((tail mean-head mean) × tail DNA %). A minimum of 50–200 cells per sample are assessed, and the DNA fragmentation for each sample is presented as an average tail DNA %, tail length, tail moment and/or olive tail moment. 

COMET can be performed under either neutral or alkaline conditions. Neutral COMET assesses only DSBs as these conditions do not support the unwinding of the DNA double helix structure. Therefore, all fragments in the COMET tail must have breaks on both strands of the DNA [133]. As only DSBs can be analysed, neutral COMET is often more subtle in its determination of SDF. One study found that the neutral COMET assay had no predictive power of fertility in men, whereas the alkaline COMET assay was the best at predicting male fertility, followed by other assays like TUNEL and SCSA [134]. Alkaline COMET is often preferred as all DNA fragmentation can be assessed, and the outcome is more robust than neutral COMET, allowing for greater differentiation of DNA fragmentation between groups [135]. Neutral and alkaline COMET can be used in tandem to determine the level of all SSBs and DSBs and thus ascertain the proportion of SSBs alone. However, the accuracy of this has yet to be confirmed. 

As sperm cell chromatin is highly compacted, DNA is often harder to access through traditional somatic cell COMET assays. The alteration of various lysis and denaturation methods has allowed for more accurate COMET assay results. However, more optimisations may be required to assess the DNA fragmentation across many species (Table 2). COMET is not widely employed as it is time-consuming to prepare and perform as described in Table 1. However, it is highly regarded for its simplicity and visualisation of DNA breakage [135], and it facilitates the detection of DNA fragmentation in cells that may be obtained with a smaller number of cells than required from other assays [136]. 

The COMET assay has provided evidence of DNA integrity’s significant impact on human pregnancy, with SDF > 50% correlating with reduced pregnancy success following IVF [110]. However, due to its downfalls, the use of either neutral or alkaline COMET assay is minimal, and there are no strong conclusions regarding fertility (Table 2). The COMET assay has been predominantly employed to measure sperm DNA integrity in bulls, where findings often indicate no significant correlation between SDF and high- and low-fertility groups [120]. The studies in Table 2 that reported a significant impact of SDF on field fertility, or comet tail length negatively affecting fertility, had very small sample sizes of five [113,114] and three bulls [112] (Table 2). The limitations of the COMET assay described in Table 1 hinder its use in both human and livestock research as a measure of sperm DNA integrity, as evidenced by the lack of literature employing this assay.

### 3.3. Transferase dUTP Nick End Labelling (TUNEL)

TUNEL detects DNA strand breaks and apoptosis in cells by labelling free 3′-OH termini on single- or double-stranded DNA [137]. TUNEL utilises the action of terminal deoxynucleotidyl transferase, which catalyses the addition of deoxyribonucleotides, specifically deoxyuridine triphosphate (dUTP), to the 3′-OH termini of DNA strands of either fragment that break off during apoptosis or strand breaks that have formed through other processes [131]. The degree of fluorescent or chemical labelling attached to the dUTP is directly proportional to the number of DNA strand breaks within the DNA. The TUNEL assay is valued for its high predictive value and potential to distinguish sub-fertile populations by identifying elevated sperm DNA fragmentation [137] (Table 1). Because of this, the TUNEL assay has been utilised in studies across species, as seen in Table 2, which have found a significant impact of DNA integrity on embryo development and fertilisation [95,96,98]. Moreover, TUNEL is a direct measure of SSBs and DSBs by incorporating modified nucleotides directly onto the site of damage [131]. The assay has the capacity to assess 10,000 cells using flow cytometry [131], facilitating efficiency and ease of use (Table 1). 

In humans, an overarching negative correlation exists between DNA fragmentation measured by TUNEL and fertilisation and pregnancy rates (Table 2). However, these results are across various ART methods, including IVF, intrauterine insemination (IUI) and ICSI, and DNA integrity measures. This may be why we see variation in results, with some studies in Table 2 using TUNEL. The several livestock studies using TUNEL, as mentioned in Table 2, have small sample sizes, limiting their robustness and complicating comparisons with the many human studies using the same assay. While the TUNEL assay is a valuable diagnostic tool for identifying sperm DNA fragmentation, the lack of standardisation and validation across laboratories hinders widespread application [131]. Encouraging the use of TUNEL in the livestock industry or production settings may require additional research employing this assay to create a concrete understanding of the impact of DNA fragmentation on livestock fertility. 

### 3.4. Chromomycin A3 (CMA_3_)

CMA_3_ is an indirect assay used to evaluate the degree of sperm chromatin protamine deficiency and is a fluorochrome specific for guanosine cytosine-rich sequences. CMA_3_ allows for indirect visualisation of protamine-deficient and decondensed sperm DNA by competing with protamine binding sites and facilitating the identification of endogenous nicks in decondensed abnormal spermatozoa with CMA_3_-positive nuclei [60,138]. Moreover, this method is directly related to the degree of protamination in mature spermatozoa [139]. Hence, this assay is useful in identifying abnormalities in histone–protamine displacement, identifiable by the bright yellow staining observed under a fluorescent microscope [140].

This technique has potential as a good predictor of fertility, whereby the percentage of staining of CMA_3_ is negatively associated with the quality of spermatozoa. This was observed in [139] in men, where samples exhibiting a high threshold of >49–77% CMA_3_ staining achieved significantly lower fertilisation rates than samples exhibiting low (8–62%) CMA_3_ staining. This result was repeated in another study, which found a negative correlation between the percentage of CMA_3_ and IVF rate and a significant difference in the mean percentage of CMA_3_-positive spermatozoa between fertile and infertile groups of men [118]. 

Regarding livestock, CMA_3_ has predominantly been used to stain bull spermatozoa to assess sperm quality, as shown by the studies presented in Table 2. A significant difference in the percent of CMA_3_ was found between bull groups, representing higher protamine deficiency in the high-fertility group [120]. However, [120] proposed that protamine deficiency in cattle may not significantly result in DNA damage, as there was no difference between fertility groups when an alkaline COMET test measured DNA fragmentation. CMA_3_ is frequently used in conjunction with another DNA integrity assay to determine fragmentation or assess sperm protamine content [119,120]. Due to this reliance on complementary assays and the uncertainties arising from limited studies in animals, CMA_3_ may be less suitable for widespread use in industry. Nevertheless, further research across various livestock species using CMA_3_ could position it as a useful tool for evaluating male fertility before ART use, akin to its use in humans.

### 3.5. Toluidine Blue (TB)

The TB test indirectly identifies DNA fragmentation in spermatozoa. The TB metachromatic dye is a sensitive external agent that incorporates into the damaged dense chromatin and binds to phosphate groups of DNA strands, resulting in chromatin staining [141]. This assay is well regarded for its simplicity and usefulness in determining sperm DNA integrity (Table 1). Moreover, it was found that a threshold of 45% is highly predictive of male infertility [116]. Despite a couple of studies finding higher TB staining in infertile groups of males, the TB test seems to be less commonly used in human research [115,142]. This may be due to the constraint posed by its ability to assess only a limited number of sperm and its labour-intensive nature [109].

TB has been used in livestock studies to identify chromatin alterations or nuclear morphology of spermatozoa, often focusing on the correlation between semen quality parameters. A study in bulls using TB found that sperm presenting chromatin instability had a larger sperm head area [143]. The same study used another assay, CMA_3_, to find that sperm with abnormal chromatin compaction did not impact early embryonic development. In a study on 20 bulls, a significant reduction in chromatin maturity was identified in sperm from low-fertility bulls compared to high-fertility bulls [144]. Another study reported a similar result, noting lower overall chromatin alterations in sub-fertile bulls compared to fertile bulls using TB [117], although this result was obtained by a small sample size of only three bulls (Table 2). Besides these studies on bulls, TB is not frequently employed in other livestock species. Instead, it is often used in combination to identify sperm morphological structure [145,146]. Unlike in human research, TB is not a common assay used in livestock solely to measure DNA fragmentation and its impact on fertility outcomes.

### 3.6. Sperm Chromatin Dispersion (SCD) Test 

The SCD test, or HALO, is a quantitative measure of DNA integrity by assessing chromatin stability based on induced decondensation [85]. Within this assay, spermatozoa are subjected to a protein depletion treatment, and the assay relies on the response of fragmented or unfragmented sperm DNA by lysis [17]. If the sperm DNA molecule is immensely broken, most of the genome will be denatured, whereas non-fragmented DNA will remain intact [147]. Following this, sperm are immersed in a lysing solution to remove protamines, which results in the spreading of DNA loops into the surrounding microgel [147]. These loops constitute a peripheral halo of DNA chromatin emerging from a central core or residual nuclear area [147]. Following the treatment, the absence of significant denaturation of sperm DNA indicates a lack of fragmented DNA, which is distinguished by the large halos of dispersed DNA representing the sperm %DFI [17]. Sperm nucleoids that have been denatured in the previous solution will appear without a halo of dispersed chromatin or to a limited extent [17]. 

SCD also contains a species-specific lysing solution that simultaneously preserves sperm morphology while facilitating the discrimination of sperm from other cell types within an ejaculate or biopsy [147]. The Halo-sperm kit (Halotech DNA, Fuencarral-El Pardo, Madrid, Spain) is widely used for the SCD test as it provides all the necessary reagents required for the assay and facilitates simplicity of use, repeatability, and consistency in results [147] (Table 1). In humans, the Halo-sperm kit has been employed to determine the level of DNA fragmentation in semen samples, with numerous studies in Table 2 showing a negative correlation between %DFI and pregnancy outcomes [86,87].

Due to the simplicity and efficiency of this test, it is extensively used in research across various animal species (Table 2). Several studies in humans have explored DNA fragmentation’s impact on embryo development and fertilisation using the SCD assay. In [148], an SDF rate of >30% resulted in a decreased quality of embryo formation, corresponding to [88] finding a decrease in eight-cell embryos and blastocysts formed with DFI > 30%. Further human research has shown a high %DFI to cause failed fertilisation and pregnancy [89,90,91]. Although these findings seem conclusive, a study found the opposite effect of high DNA fragmentation causing increased fertilisation rates in humans [18]. 

Similar findings have been seen in other species, including rams, with some studies reporting high SDF in high-fertility groups [92] or no significant impact of SDF on fertility measurements [19] (Table 2). However, there is an equal level of research reporting contradicting results, with the SCD assay identifying SDF as being higher in the low-fertility group of rams than in the high-fertility group [92]. In Holstein bulls, a study found a significant impact of SDF, negatively impacting estimated breeding value and estimated genetic value [93]. Although the research using SCD in livestock is limited compared to humans (Table 2), these results impacting breeding and genetic value are important factors in producers’ income. This may act as an incentive for producers to employ DNA integrity assessments in investigating male fertility before implementing ART programmes.

### 3.7. 8-Hydroxyguanine

8-Hydroxyguanine (8-oxoG) is the most common measurement of oxidative DNA damage. It involves measuring 8-oxoG, mainly produced by ROS-inflicted damage [149], in digested DNA using high-performance lipid chromatography (HPLC) with electrochemical detection to determine the level of 8-oxoG [150]. 8-oxoG levels in different biological fluids have been investigated as oxidative stress marker candidates, including urine [150,151], saliva [152,153] and semen. However, its use as an assay to measure oxidative DNA damage in sperm is quite limited, with only two studies investigating this to our knowledge [154,155]. One of these studies found 8-oxoG to be linked with human male infertility, with a group of 91 infertile males having significantly elevated levels of 8-oxoG than the control group (normozoospermic) of 32 males [154]. This study also found statistically significant correlations between the level of 8-oxoG and seminal parameters, including concentration, motility and morphology. The other study found a similar finding in bulls, with one bull classified as infertile due to asthenoteratozoospermia having significantly higher levels of 8-oxoG than a group of fertile bulls [155]. Further research is now needed to expand the sample size and corroborate its ability to be used as a marker of DNA damage in sperm and thus a predictor of fertility. 

## 4. Implications for the Livestock Artificial Breeding Industry 

Despite the multiple assays discussed, the analysis of DNA integrity remains an uncommon part of standard semen assessment in animal industries. Although its incorporation into human fertility research has yielded a substantial understanding of sample quality and viability, commercial adoption remains limited. This is primarily due to the uncertain clinical value or relationship between DNA integrity and successful IVF or ARTs. While DNA damage is broadly associated with male infertility, its direct impact on fertility outcomes following ARTs lacks conclusive evidence, with studies listed in Table 2 reporting conflicting findings. Additionally, there is an inconclusive understanding amongst researchers on whether DNA integrity assays offer novel insights beyond existing semen assessment parameters. Until validated DNA integrity assays offer unique fertility insights, there will be a preference for current simple and cost-effective tests like sperm motility and kinematics, morphology and concentration assessment. Many reviewed DNA assays necessitate expensive equipment like flow cytometry and specialised training for accurate use and interpretation (Table 1). The subjective nature of microscopy protocols in certain assays presents challenges in interpretation, resulting in inconsistent findings within the same assay and species shown in Table 2. The lack of conclusive findings regarding fertility and DNA integrity from each assay due to these issues underscores the uncertainty about identifying the most suitable assay and contributes to the hesitance in the commercial industry to adopt DNA integrity as a semen quality parameter. Incorporating DNA integrity assessment into routine commercial practice will remain challenging until standardised protocols and validated assays for specific species’ DNA integrity are established. Ultimately, until the above-mentioned DNA assays can be assessed side by side in the same study, using identical samples and incorporating large-scale fertility data, it will be difficult to determine the most accurate assay correlated with field fertility or ART success. Given the variability surrounding what each assay measures within DNA and when and how, assays must be compared reliably within the same studies on the same samples to make an informed decision for industry.

Despite the uncertainties surrounding DNA integrity assay outcomes, their potential to offer unique fertility insights remains promising. Studies including [90] using SCD, [118] using CMA_3_, [99] using TUNEL, and others in Table 2 have documented a significant negative impact of high DNA fragmentation levels on fertility outcomes. If substantiated with larger sample sizes and consistent findings within species and assays, this emerging trend could have significant implications for both the animal breeding and human fertility industries. Among the assays, the SCD test through the use of the Halo-sperm kit appears as the most promising assay for assessing DNA integrity in livestock industry practices, providing results comparable to the commonly used SCSA without the expensive equipment and expertise required. The accurate prediction of DNA integrity in semen can contribute significantly to the quality assurance of samples used in ART programmes. Advanced semen assessment for the precise prediction of sire fertility before insemination has the potential to substantially enhance success rates by excluding sires with sub-optimal fertility results. As ART procedures become more reliable and consistently successful, the prospect of increased industry adoption leads to improved genetic gain and overall advancements in reproductive technologies.

## 5. Conclusions

The escalating prominence of ART use in livestock and human contexts reflects a response to infertility challenges and a means to enhance reproductive outcomes. Recent advances in animal research, particularly in the context of AI programmes, highlight the variability in success outcomes linked to semen quality factors. Human semen assessment, a precursor to ART utilisation, is evolving from subjective evaluations to incorporate standardised objective technologies. Despite extensive investigations into the impact of semen factors on fertility across various livestock species, the influence of DNA fragmentation remains inconclusive and is yet to be integrated into standard semen analysis of animals. There is, however, growing evidence that an increase in DNA fragmentation negatively impacts reproductive outcomes in livestock studies. By improving consistency and standardisation in the methodology used across studies while also increasing sample sizes, this trend may strengthen in future studies. Introducing DNA integrity assays into the semen assessment toolbox for livestock species could increase the accuracy of pre-screening assays, helping to reduce the use of poor-performing sires or samples and therefore increasing the success rates of ARTs, enhancing genetic gain and production traits for livestock industries.

## Figures and Tables

**Table 1:** Overview of DNA integrity assessment assays in spermatozoa. Abbreviations: SCSA, Sperm Chromatin Structure Assay; TUNEL, Transferase dUTP Nick End Labelling; SCD, Sperm Chromatin Dispersion; COMET, single-cell gel electrophoresis; TB, toluidine blue; CMA_3_, chromomycin A3; DNA, deoxynucleic acid; ss-DNA, single-stranded DNA; ds-DNA, double-stranded DNA; SSB, single-stranded break; DSB, double-stranded break.

Assay	Predominant Species	Equipment	Technique	Advantages	Limitations
SCSA	Human, bull, ram, boar, and stallion	Flow cytometry	Measures susceptibility of sperm nuclear DNA to acid-induced denaturation.	Efficient, quantitative assessment. Has shown consistency in results.	Expensive equipment (flow cytometry) and skilled interpretation and training needed.
TUNEL	Human, bull, stallion	Flow cytometry or microscopy	Labels free 3′-OH termini on ss-DNA or ds-DNA to detect DNA strand breaks and apoptosis.	High predictive value. Direct, easy to use and efficient.	Expensive equipment if flow cytometry used. Skilled equipment training needed.
SCD	Human, ram, bull	Microscopy	Sperm subject to protein depletion treatment and assay relies on response of fragmented or unfragmented sperm DNA by lysis.	Simultaneously preserves sperm morphology. Simple and efficient. No expensive equipment cost.	Observer subjectivity
COMET	Human, bull	Microscopy	Assesses level of SSB or DSB in a cell following agarose cell suspension, cell lysis, DNA denaturation, electrophoresis and microscopy.	Can be quantified to determine cells’ degree of DNA fragmentation. Can be used on a smaller population of cells.	Time-consuming. High level of training required to use specific computer programmes. No strong conclusions regarding fertility.
TB	Human, bull	Microscopy	Metachromatic dye binds to damaged dense chromatin and phosphate groups of DNA strands.	Simple and inexpensive. Highly predictive of human fertility.	Labour intensive. Can only access a limited number of sperm cells. Observer subjectivity.
CMA_3_	Human, bull	Flow cytometry or microscopy	CMA_3_ compete for protamine binding sites to identify endogenous nicks in decondensed abnormal sperm.	Directly related to degree of protamination. Can identify abnormalities in histone–protamine displacement.	Observer subjectivity with microscopy. Expensive equipment if flow cytometry used.

**Table 2:** The predictive power of DNA integrity assessment methods and their link to fertility across species. Abbreviations: SCSA, Sperm Chromatin Structure Assay; AO, acridine orange; SCD, Sperm Chromatin Dispersion test; COMET, single-cell gel electrophoresis/comet assay; TUNEL, terminal deoxynucleotidyl Transferase dUTP Nick End Labelling; CMA_3_, chromomycin A3; TB, toluidine blue; AB, Aniline Blue; SDF, sperm DNA fragmentation; DFI, DNA Fragmentation Index; HDS, high DNA stainability; TH, threshold; SE, sensitivity; SP, specificity; AUCROC, area under receiver operating characteristic curve; OR, odds ratio; 95% CI, 95% confidence interval; PPV, positive predictive value; NPV, negative predictive value; PLR, positive likelihood ratio; NLR, negative likelihood ratio; NRR, non-return rate; CP, clinical pregnancy; LB, live birth; CR, conception rate; FR, fertilisation rate; SU, swim-up; OO, own oocyte; DO, donated oocyte; ART, assisted reproductive technology; IVP, in vitro embryo production; IVF, in vitro fertilisation; ICSI, intracytoplasmic sperm injection; IUI, intrauterine insemination; AI, artificial insemination; AIH, artificial insemination by husband; ET, embryo transfer; PD, protamine deficiency; HF, high fertility; LF, low fertility; ESBV, estimated bull service value; EGV, estimated genetic value; PR, pregnancy rate; DNA, deoxynucleic acid; NDS, no significant difference; NS, not significant; SD, significant difference; COMP αt, cells outside the main population; H-DNA, head DNA density; T-DNA, tail DNA % intensity; TM, tail moment (% T-DNA × Tail length); OTM, olive tail moment.

Species	ART Method	Sperm DNA Integrity Measure	Correlation/Outcome	Reference
SCA				
Human (*n* = 165)	ICSI	DFI	DFI lower when pregnancy was achieved (14.86%) than when no embryonic heartbeat detected (17.37%); *p* = 0.031 Low DFI in spermatozoa corresponded with faster embryo development to reach the blastocyst stage DFI positively correlated with a delay in 8 out of 13 embryonic development periods	[86]
Human (*n* = 420)	ICSI (OO or DO)	SDF before and after SU of fresh and frozen–thawed sperm	SDF increase of 10%; probability of negative pregnancy outcome increased by 1.31 SDF affects pregnancy outcome (OR 0.973, 95% CI 0.948–0.999, R^2^ 0.069; *p* = 0.037)	[87]
Human (*n* = 377)	ICSI	DFI >30% (mean 39.25)	DFI > 30% decreased number of 8-cell embryos on day 3 (3.97) Number of blastocysts formed on day 5 (1.6), CP (23.13%) and LB (13.43%) from <30% DFI (5.96, 2.44, 35.83%, 28.75%; *n* = 237); (*p* = 0.001, *p* = 0.001, *p* = 0.05, *p* = 0.005) Miscarriage rate (9.7%) increased from <30% DFI (7.08%); *p* = 0.005	[88]
Human (*n* = 135)	ICSI	SDF > 22.3%	SDF > 22.3% FR (55.1%) lower than sperm > 22.3% SDF (74.9%); *p* < 0.001. SDF negatively correlates with FR (r = −0.433; *p* < 0.001).	[89]
Human (*n* = 94)	ICSI	Low DFI (<15%); (*n* = 50) Mod DFI (15–30%); (*n* = 31) High DFI (>30%); (*n* = 13)	High-DFI group was unable to achieve pregnancy following ICSI.	[90]
Human (*n* = 85)	IVF/ICSI	SDF	SDF negatively correlated with FR (r = −0.241; *p* = 0.045) and implantation rate (r = −0.25; *p* = 0.042) of samples assessed post-capacitation Asymmetrical nuclei more frequent with increased SDF in capacitated sperm; *p* < 0.05 Higher SDF lowered ability to develop expanded blastocyts on d6; *p* < 0.05	[91]
Human (*n* = 867)	IVF (*n* = 379)	Low DFI (<30%); (*n* = 343) High DFI (>30%); (*n* = 36)	FR higher in High-DFI group (86.9%) than Low DFI (78.4%); *p* < 0.05 CP lower in High-DFI group (25%) than Low DFI (48.6%) *p* < 0.05	[18]
Rasa Aragonesa ram (*n* = 8)	Field fertility odds ratio 1.4–1.7 (HF; *n* = 4) 0.6–0.9 (LF; *n* = 4)	SDF	SDF following 6 and 24 h incubation at 37 °C higher in LF (20.27%, 31.24%) than HF (14.42%, 22.32%), *p* < 0.05, *p* < 0.01	[92]
Holstein bull (*n* = 201)	AI (≈533/bull)	SDF	SDF following 0 h incubation negatively correlates with ESBV and EGV (r = −0.45, r = −0.36; *p* < 0.0001) SDF following 6 h incubation negatively correlates with ESBV and EGV (r = −0.49, r = −0.38; *p* < 0.0001)	[93]
Stallion (*n* = 11) Moderate fertility = PR <50% (*n* = 8) Good fertility = PR >50% (*n* = 3)	Uterine AI (catheter)	SDF in semen cool-stored in spring vs. summer	Negative correlation between SDF and PR (r = −0.619; *p* < 0.001) Moderate-fertility group has higher SDF at 0 (7.9) and 6 h (15.4) cooled storage compared to good-fertility group (3.83%, 9.58%); *p* < 0.05 SDF rate higher in sperm cool-stored in summer than in spring and PR lower in summer than spring; *p* < 0.05	[94]
TUNEL				
Human (*n* = 105)	ICSI	DFI > 20% (% TUNEL positive)	DFI > 20% decreased the number of good-quality embryos (6.63), implantation rate (4.9%), and number of pregnancies (3) from <20% DFI (11, 15.79%, 9); *p* = 0.018, *p* = 0.002, *p* = 0.046	[95]
Human (*n* = 36)	ICSI	SDF	SDF negatively correlated with mean total embryo score (r = −0.64, *p* < 0.001) and mean transferred embryo score (r = −0.63, *p* < 0.001) SDF TH 17.6% is predictive of pregnancy (*p* < 0.021)	[96]
Human (*n* = 303)	IVF/ICSI	SDF	SDF higher in ICSI (6.8%) than IVF (1.9%) group (*p* < 0.05) SDF negatively correlated with IVF and ICSI FR (r = −0.357; *p* < 0.001, r = −0.222; *p* = 0.04) Good embryo rate (*p* < 0.05) SDF <4% (46.4%) higher than 10–15% SDF (31.6%) in IVF group SDF <4% (45.6%) higher than 10–15% SDF (33.0%) in all samples	[97]
Human (*n* = 45)	IVF	SDF (%TUNEL positive)	SDF negatively correlated with FR; *p* < 0.05 >55% SDF resulted in lower FR than <35% SDF; *p* < 0.05	[98]
Human (*n* = 68)	ICSI	SDF (%TUNEL positive)	SDF higher in non-pregnant group than pregnant group	[98]
Holstein bulls (*n* = 5)	AI	SDF (% TUNEL positive)	SDF higher in LF (below-average fertility) bulls (20–25%) than average or HF (above-average fertility) bulls (<15%); *p* < 0.05	[99]
Norwegian red bulls (*n* = 30)	AI	SDF (% TUNEL positive) 4.8–9.4% 9.4–21.2%	2.2–4.8% SDF 10% significantly higher odds of AI success (*p* = 0.006)	[100]
SCSA				
Human (*n* = 2262)	ART (AIH-IUI *n* = 1185, IVF *n* = 1221, ICSI *n* = 216)	High DFI (≥30%) Med DFI (15–30%) Low DFI (≤15%)	AIH-IUI: Early abortion rate increased in High-DFI (27.3%) and Med-DFI (14.6%) groups compared to Low DFI (4.9%); *p* < 0.05	[101]
Human (*n* = 1316)	IVF	DFI >11.3%	DFI higher in non-pregnant (17%) than pregnant group (14.9%); *p* = 0.001 DFI is a predictor of pregnancy outcome (*p* = 0.023) DFI TH > 11.3% is predictive of pregnancy outcome (AUC^ROC^ 0.574, 95% CI 0.541–0.607, SE 56.1%, SP 60%, PPV 77.9%, NPV 35.1%)	[102]
Human (*n* = 266)	ICSI	DFI >30.3%	DFI higher in non-pregnant (31.5%) than pregnant group (26.3%); *p* = 0.01 DFI is a predictor of pregnancy outcome (*p* = 0.004) DFI TH > 30.3% is predictive of pregnancy outcome (AUC^ROC^ 0.567, 95% CI 0.487–0.647, SE 50.6%, SP 68.8%, PPV 79.3%, NPV 37.0%)	[102]
Human (*n* = 96)	ICSI (*n* = 155)	19% DFI TH	DFI TH ≥ 18–19% predicts the outcome of ICSI (*p* < 0.005) DFI negatively correlates with continuing pregnancies (r = −0.184, *p* = 0.022), and positively correlates with non-pregnancy (r = 0.197, *p* = 0.014) Continuing pregnancy rate and implantation rate lower in ≥19% DFI group (14.9%, 12.1%) than <19% DFI (34.6%, 27.2%); *p* = 0.005, 0.001 Non-pregnancy rate significantly higher in ≥19% DFI group (75.7%) than <19% DFI (55.6%) *p* = 0.008	[103]
Ram (*n* = 15)	Vaginal AI	Mean DFI and heterogeneity (SD DFI) of SDF in the total sperm population	Mean DFI negatively associated with 25 d NRR (OR 0.98, 95% CI 0.97–1, *p* = 0.039) SD DFI negatively associated with 25 d NRR (OR 0.98, 95% CI 0.97–0.99, *p* = 0.001	[16]
Finnish Ayrshire bull (*n* = 43) >55% 60 d NRR (F; *n* = 21) <55% 60 d NRR (SF; *n* = 22)	AI (*n* ≈ 5964/bull)	DFI SD-DFI HDS	HDS higher in F (0.61%) than SF bulls (0.48%); *p* < 0.05 HDS positively correlated with calving rate (r = 0.31; *p* < 0.05)	[12]
Norwegian red bull (HF; *n* = 19) (LF; *n* = 18)	AI	DFI HDS	HF bulls had lower DFI and HDS (1.84%, 2.93%) than LF bulls (3.5%, 4.31%) *p* < 0.01 DFI and HDS negatively correlate with 56 d NRR56 (r = −0.57, *p* = 0.0003, r = −0.37, *p* = 0.026) DFI significantly predicts 56 d NRR (*p* < 0.01)	[104]
Norwegian red bulls (*n* = 30)	AI	DFI	7.5–21.6% DFI reduced odds of AI success from average (6%; *p* = 0.011) 1.6–3.8% DFI increased odds of AI success from average (7%; *p* = 0.010)	[100]
Swedish red bull (*n* = 14) Holstein bull (*n* = 6)	AI	DFI 3.31% TH Below-average 56 d NRR (BAB, *n* = 5) Average 56 d NRR (AB, *n* = 9) Above-average 56 d NRR (AAB, *n* = 6)	DFI decreased in AAB (2.88%) compared to BAB and AB (6.23%, 4.65%); *p* < 0.05 DFI negatively correlated with adjusted 56 d NRR (r = −0.61; *p* = 0.01) DFI can differentiate between BAB and AAB (R^2^ = 0.56; *p* = 0.02) DFI TH 3.31% accurately predicts 56 d NRR (SE 66.7%, SP 100%, AUC^ROC^ 0.8)	[11]
Holstein bulls (*n* = 20)	AI	DFI (COMP *α_t_*)	DFI negatively correlated to NRR (r = −0.60 *p* < 0.01) DFI lower in mature bulls than in young bulls (*p* < 0.01)	[105]
Holstein bulls (*n* = 19)	AI (*n* = 192)	SDF	SDF negatively correlated with 56 d NRR (r = −0.287, r^2^ = 0.082; *p* < 0.05)	[106]
Bulls: Holstein (*n* = 156) Jersey (*n* = 39)	AI (*n* = 75,610)	DFI SD-DFI HDS	Sperm without DFI (97.5%) predicts 56 d NRR (*p* < 0.0001) Sperm with moderate DFI (2.4%) predicts 56 d NRR (*p* < 0.0001) Sperm with High DFI (0.2%) predicts 56 d NRR (*p* < 0.0003) SD-DFI (33.3%) predicts 56 d NRR (*p* < 0.0001) HDS (2.8%) predicts 56 d NRR (*p* < 0.0004)	[107]
Boar (*n* = 18)	AI	Mean DFI SD DFI of SDF in the total sperm population	DFI negatively correlated with farrowing rate (r = −0.55, *p* < 0.01) and ANB (r = −0.54, *p* < 0.01) SD DFI negatively correlated with farrowing rate (r = −0.67, *p* < 0.002) and ANB (r = −0.54, *p* < 0.02) DFI TH 6% is predictive of farrowing rate and ANB (OR 1.5, 95% CI 1.21–1.94, *p* = 0.0003, SE 83%) SD DFI TH 40 is predictive of farrowing rate and ANB (OR 2.5, 95% CI 1.87–3.32, *p* = 0.001, SE 92%)	[15]
Boar (*n* = 160)	AI	DFI > 3% DFI > 2.1%	DFI > 3% (0 h storage) reduced ANB/litter (13.9) from DFI < 3% (14.87–14.94); *p* < 0.01 For Landrace and Danish Large White boars, DFI > 2.1% after 24 h 18 °C storage had lower litter size (14.4, 14.2) than DFI < 2.1% (15.1, 15.1); *p* < 0.01	[108]
Stallion (*n* = 41)	AI	DFI SD DFI Mean DFI	DFI negatively correlated with PR (r = −0.63, *p* < 0.05)	[109]
COMET				
Human (*n* = 339)	IVF (*n* = 203) ICSI (*n* = 136)	>50% SDF (Comet Score) TH	SDF higher in non-pregnant group than LB or miscarriage groups (*p* < 0.05) following IVF SDF TH > 50% reduced pregnancy (16.2, *p* = 0.005) and LB rates (13.1%, *p* = 0.007) following IVF	[110]
Bulls (*n* = 45)	IVP (-ET)	Mean-DNA Mean H-DNA T-DNA	T-DNA higher in group 4 (8.53%) than group 3 (4.31%); *p* < 0.05 Mean-DNA and Mean H-DNA negatively correlated with blastocyst rate (r = −0.375, r = 0.389; *p* = 0.02, *p* = 0.016)	[111]
Italian Mediterranean Buffalo bulls (*n* = 3)	AI (*n* = 528)	% H-DNA % T-DNA TM OTM	% H-DNA TH ≥ 86% (and its relative % T-DNA < 14%) predicts successful d30/45 pregnancy (AUC^ROC^ 0.56, SE 81%, SP 26%; *p* < 0.05) Tail area TH ≤ 58 μm^2^ predicts successful d30/45 pregnancy (AUC^ROC^ 0.56, SE 80%, SP 26%; *p* < 0.05)	[112]
Nili–Ravi Water Buffalo bull (*n* = 5)	AI (*n* = 514)	Comet length, % H-DNA, %T-DNA, tail length, TM, OTM	Tail length negatively correlated with fertility rate (r = −0.7; *p* = 0.04)	[113]
Buffalo bulls (*n* = 6) (HF; *n* = 3) (LF; *n* = 3)	IVP (-ET)	SDF (% of cells comet tail+)	SDF higher in LF group (18.72%) than HF group (8.94%; *p* < 0.05).	[114]
TB				
Humans (*n* = 1386)	Infertile and normospermic	SDF%	Mean TB staining was higher in infertile group than normospermic (*p* = 0.005)	[115]
Human (*n* = 142)	Infertile and fertile men	%TB dark cells %TB light cells	TB dark cells and light cells had 92% and 90% specificity, respectively, for predicting infertility Both poor predictors of fertility (42 and 32% sensitivity, respectively)	[116]
Bull (*n* = 8)	Fertile and sub-fertile (subjects to scrotal insulation)	Chromatin alteration types (Base, Basal half, Central axis, Dispersed and Whole)	Greater (*p* < 0.01) chromatin decondensation and heterogeneity were recorded in sub-fertile bulls	[117]
CMA_3_				
Human (*n* = 139)	IVF	%CMA_3_ positivity	%CMA_3_ positivity has negative correlation with farrowing rate %CMA_3_ positivity significant difference between fertilising and non-fertilising patients.	[118]
Human (*n* = 30)	ICSI	%CMA3	%CMA_3_ positivity showed significant negative correlation with FR	[119]
Bulls (*n* = 12) (140 bulls ranked on embryo development rate, chose bottom 6 and top 6)	IVP	%CMA_3_	Significant difference in %CMA_3_ between groups (*p* = 0.03), suggesting greater protamine deficiency in the high fertility group	[120]

## Data Availability

The authors confirm that there are no data to be made available for this review.
